# Supplementary figures and images for: How competition can drive allochronic divergence: A case study in the Marine Midge, Clunio marinus
Source: PLoS Comput Biol. 2026 Apr 27;22(4):e1014235. doi: 10.1371/journal.pcbi.1014235 (PMC13148829; doi:10.1371/journal.pcbi.1014235)

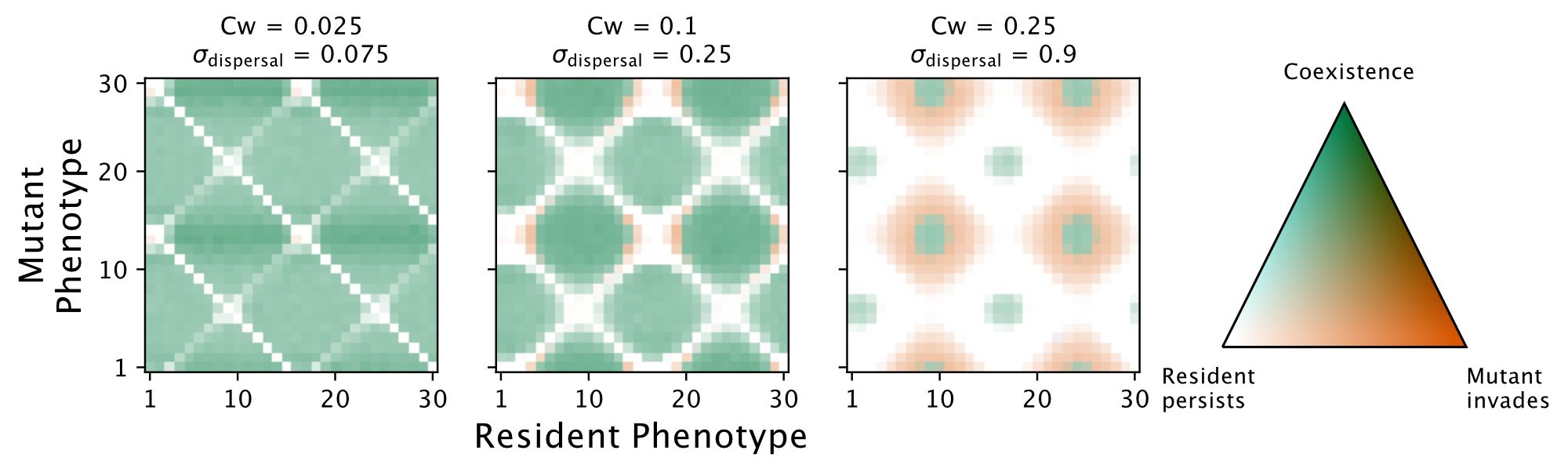

Supplement: S1 Fig — White indicates regions where the resident phenotype persists, orange indicates where the mutant phenotype would invade, and green represents regions where the resident and mutant would coexist. The value of each cell is the average of 1000 simulations. (TIFF) [file pcbi.1014235.s001.tiff]

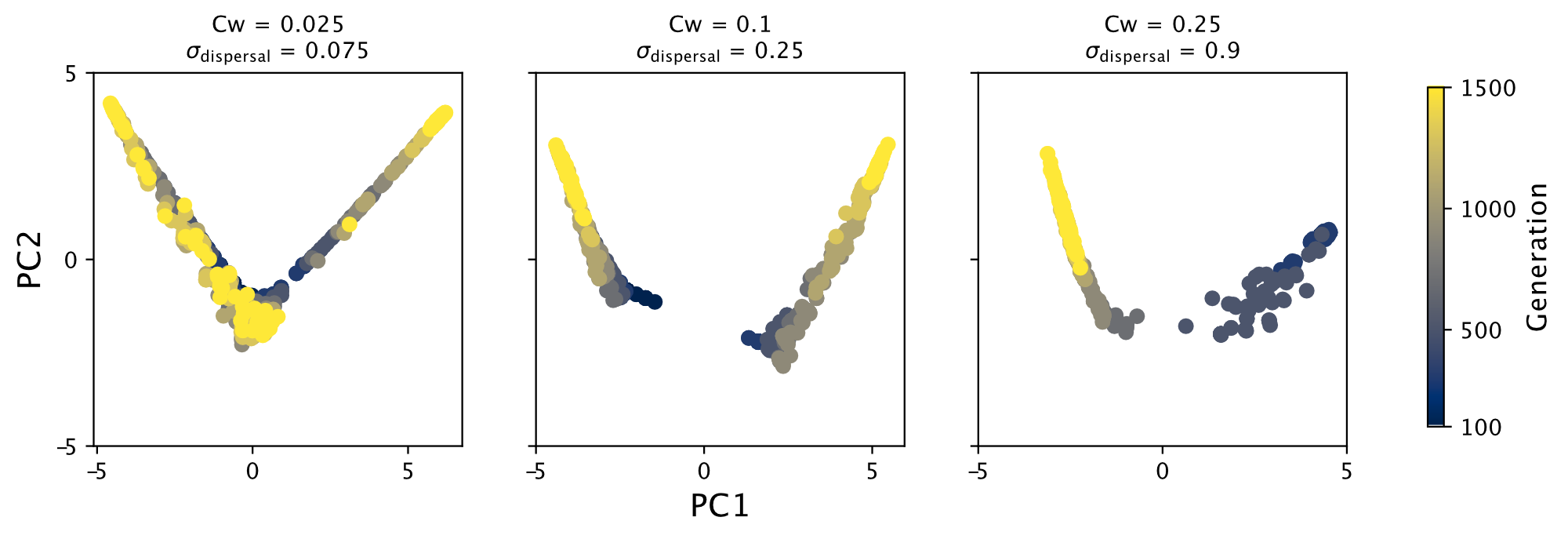

Supplement: S2 Fig — The three plots correspond to the different Cw/σdispersal combinations of the three simulation runs in Fig 4B. At low values, we find three or more genetic clusters at generation 1500 (yellow dots), though these clusters are not necessarily discrete. At intermediate values, we find two discrete clusters, while at high values, there is only one cluster at generation 1500. (TIFF) [file pcbi.1014235.s002.tiff]

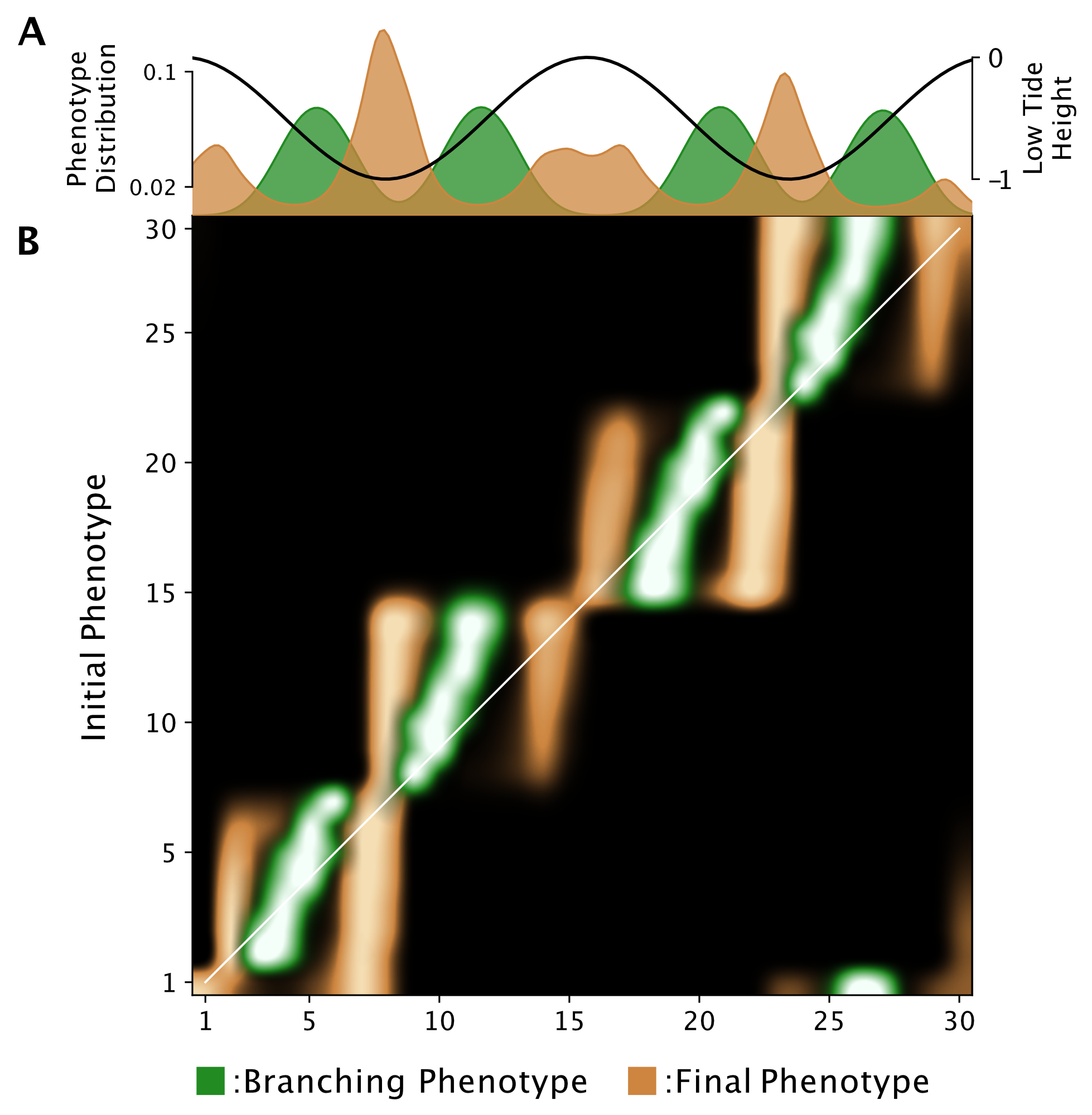

Supplement: S3 Fig — A: Kernel density estimates of the distribution of branching phenotypes (in green) and the distribution of final phenotypes (in brown). The black line indicates the height at low tide during the lunar month. B: The relationship between initial phenotype and branching/final phenotype. The diagonal (i.e., where initial phenotype = branching/final phenotype) is represented by a white line. Interpolation between cell values in the heat map has been applied to make the pattern more clear. (TIFF) [file pcbi.1014235.s003.tiff]

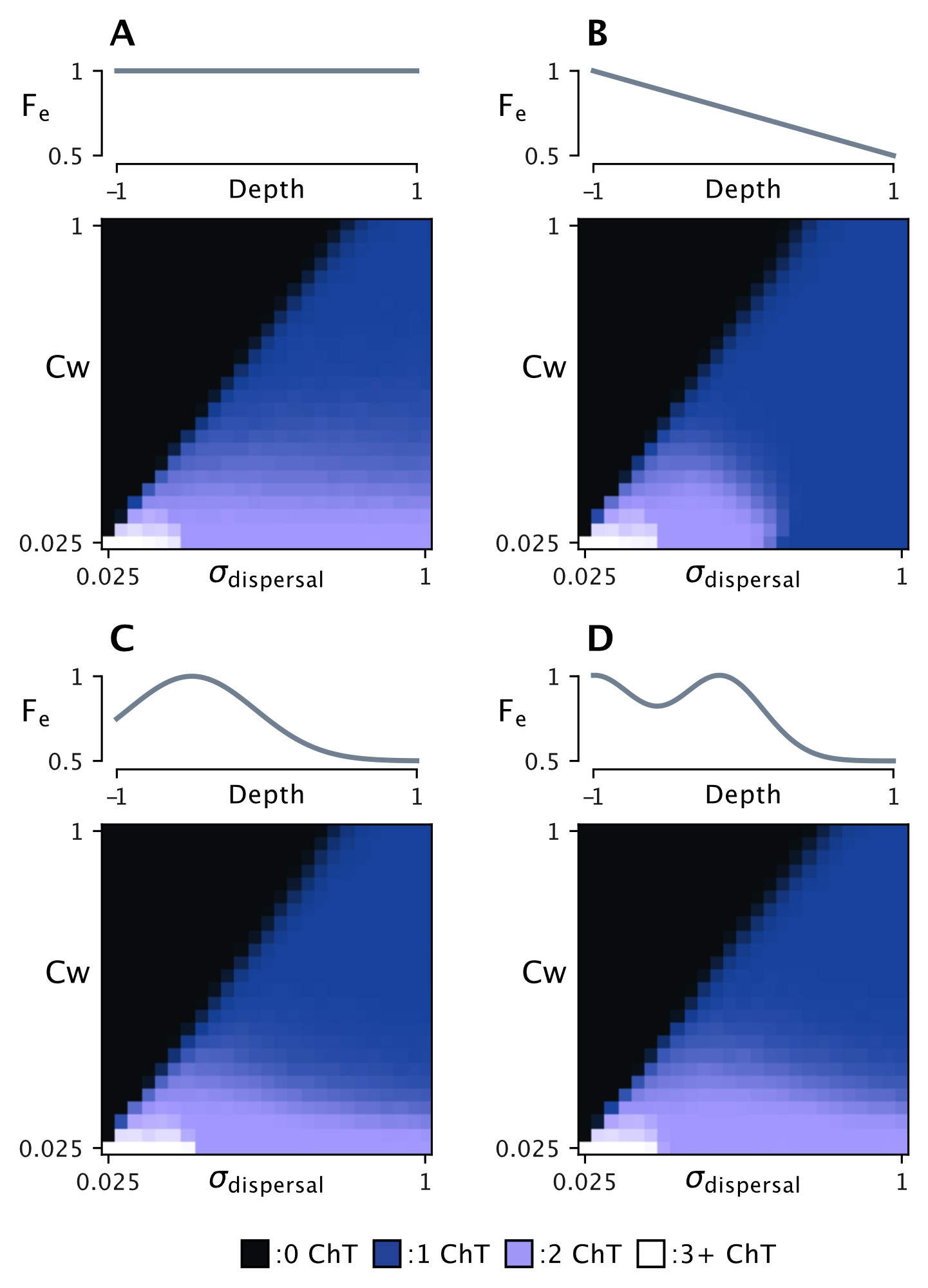

Supplement: S4 Fig — The fitness functions tested are A: a flat fitness function (i.e., no relationship between fitness and depth), B: a linear fitness function, C: a unimodal fitness function, and D: a bimodal fitness function. The average number of chronotypes for 1000 simulations at generation 1500 for each combination of the competition kernel width Cw and dispersal rate σdispersal is plotted below each fitness function. Divergence was observed (indicated by the lilac and white regions) for every fitness function tested. (TIFF) [file pcbi.1014235.s004.tiff]
