# Supplementary material for: How competition can drive allochronic divergence: A case study in the Marine Midge, Clunio marinus
Source: PLoS Comput Biol. 2026 Apr 27;22(4):e1014235. doi: 10.1371/journal.pcbi.1014235 (PMC13148829; doi:10.1371/journal.pcbi.1014235)
Supplement: Attachment — Submitted filename: response_to_reviewers.pdf [file pcbi.1014235.s006.pdf]

Notes on revisions made to manuscript Paper #PCOMPBIOL-D-25-02324

We thank the Editors for the handling of our manuscript "How Competition can Drive Allochronic Divergence: A Case Study in the Marine Midge, *Clunio marinus*" in PLOS Computational Biology. All points raised by the Editors and Reviewers 1, 2, and 3 have been fully addressed and incorporated into the revised manuscript. We are grateful to the reviewers and the editorial team for their valuable feedback and careful handling of our work, which has substantially improved the manuscript.

We thank the editors and reviewers for their carefully reading our manuscript. Below we address each of their comments point by point.

Response to Editors

Reviewer Comment 1.1 — In general, the reviewers appreciated this study and noted only a few areas of potential confusion. In particular, Reviewers 2 and 3 both made relatively minor comments on the clarity of the presentation. Reviewer 3 mentioned the structure of the first section of the results, which captures the biological background and a brief discussion of the model. Here, the authors may want to consider placing the methods before the results, as the model structure/parameterization is important for the results that follow. Similarly, the authors might consider including Tables S1 and S2 (the model parameters) in the Methods, as these parameter values are important aspects of the model. Finally, Reviewer 1 raises important questions on the model structure and justification for some modelling choices in points 2, 4, and 5. I think it is important to further explain these modelling choices before publication.

Reply: We thank the editors for the suggestions, and have moved supplemental tables 1 and 2 into the methods. We decided the keep the methods after the results, as they contain many details that are not strictly necessary to understand the results. Instead, we have updated the text to make more reference to Figure 1, which gives a graphical overview of our model.

Response to Reviewer 1

Reviewer Comment 1.2 — In line 195 where it states "an integer T which ranged from 0 to 30"., it's clear that T refers to the emergence phenotype. Using T to denote both the timing of emergence phenotype and the lunar day may lead to some confusion. I suggest using something like T_e to denote the phenotype.

Reply: In our model, T refers solely to the emergence phenotype (the lunar day in which an individual emerged), and is only used in equation 1 for calculating the depth of the low tide when an individual emerges. We have amended the methods, figure 1 caption, and figures 2, 3, and 4 to try and make it clear that T is referring specifically to the emergence phenotype.

Reviewer Comment 1.3 — In lines 202-213, it states that "changes to the emergence phenotype could only occur if the random number drawn was greater than 0.5 or less than -0.5". If $\mu = 0.5$ and $\sigma = 0.18$, the chance of getting anything less than -0.5 is incredibly low, biasing mutations towards those that increase T. I suggest the authors to either reconsider the algorithm for generating mutations or refine their explanation for how the mutations are implemented, with a mention of its propensity for increasing/decreasing T.

Reply: We thank the reviewer for catching this, as we had made a mistake in writing "greater than 0.5 or less than -0.5". We had meant to write "greater than 1 or less then 0". This has now been corrected in the text on line 492.

Reviewer Comment 1.4 — I notice that mutations may act to increase/decrease the emergence phenotype T. Please could the authors explain how they handled the scenario where a mutation occurs at a boundary case i.e. if a mutation decreasing T at $T = 0$ or increasing T at $T = 30$. It would seem unreasonable if a mutation caused T to jump all the way from 0 to 30 or vice versa.

Reply: We have provided more explanation for this in the text on line 497. In the model without recombination, we used the modulo operator on the phenotype of offspring after mutation to bound the phenotype between 1 and 30. As this model was clonal, crossing between 1 and 30 did not impact reproduction. For the model with recombination and sexual reproduction, we used the "period boundaries" function of SLiM's spatial engine to ensure that a phenotype of 0 and 30 were equivalent (see line 587 in the text). In addressing this comment, we did notice some inconsistencies in the text and figures regarding if the phenotype ranged from 0 to 30 or 1 to 30. We have now also corrected the text so that the phenotype is always from 1 to 30.

Reviewer Comment 1.5 — In Figure 3, the simulation is run for "500 generations" while in Figure 4, the simulation is run for "1500 generations" and 3000 generations in Figure 5. I would appreciate if the authors could explain why the simulation was run for a different number of generations in each model (clonal, sexual and sexual with divergence in genetic parameter space).

Reply: The reason for the differing number of simulations was due to differences in the speed at which divergence happened between the simple and extended models, as well as under certain genomic architectures. Changing the number of generations was done to ensure that simulations were not terminated prematurely, while also avoiding excessive computation. We have now added justification for why the number of simulations is different on lines 236 and 278.

Reviewer Comment 1.6 — In the environmental fitness F_e in equation 2, please provide some sentences to justify the choice of the constant 6 in e^{6D+1} as well as the + 1.

Reply: We have modified the methods:larval fitness section of the text to be more explicit about our choices for the constants of 6 and +1 in our environmental fitness function.

Reviewer Comment 1.7 — Lines 422-428 in the discussion provides a description about the parameter values where divergence occurs, however the wording makes it seems confusing e.g "$Cw = 0.25$ to $Cw = 0.025$ and $\sigma_{dispersal} = 1$". I had to refer to figure 4 to understand precisely what that meant. I recommend the authors to explicitly refer to figure 4 and rephrase it, for example "in the model with recombination (see figure 4), we observe divergence for values of (Cw,$\sigma_{dispersal}$) ranging

from (0.25,0.25) to (0.025,1)". Alternatively, the authors may consider placing this description in the results section rather than keeping it in the discussion.

Reply: We thank the reviewer for the nice suggestion and have incorporated it into the discussion. We elected not to move this statement to the results as these values are educated guesses based on the biology of *C. marinus* and the tidal ranges in Roscoff, France, rather than something that was properly quantified for our study.

Reviewer Comment 1.8 — I understand that the current model is an individual-based model. However, it would be worth mentioning somewhere in the discussion about the possibility of an alternative ODE based approach that enables some analytical reasoning (see Geritz et al 1996, Phys. Rev. lett. 10.1103/PhysRevLett.78.2024, Liu et al 2025 Plos Comp Biol Cell size and selection for stress-induced cell fusion in unicellular eukaryotes — PLOS Computational Biology). This opens up the possibility of identifying evolutionary attractors and evolutionary singularities that dictate such branching event. I realise that incorporating genetic recombination may make this challenging, but a few sentences to discuss the feasibility of such approaches would be valuable.

Reply: We thank the reviewer for noticing this. In fact, our first approach was to include a deterministic model. However, as correctly spotted by the reviewer, our keenness to include the genetic complexities forces us to reconsider the approach and hence also to target PLoS Computational Biology. Since we are also eager to derive some system-independent general principles, we have now added a few clarifications to this end as to what assumptions may make this possible in the second-to-last paragraph of the Discussion.

Reviewer Comment 1.9 — It took me a while to realise that σ_{effect} is only present in the sexually reproducing diploids model, after seeing it in Table 2. Please make this clear in section Methods:Emergence.

Reply: We thank the reviewer for pointing out the lack of clarification about this. We have updated the Methods:Emergence section to address this.

Reviewer Comment 1.10 — Line 228, typo $\sigma_{divergence}$, please correct to $\sigma_{dispersal}$.

Reply: We thank the reviewer for finding this error and have fixed it in the text.

Reviewer Comment 1.11 — Cw is denoted as C_w in places e.g. on line 427. Please choose your desired convention and correct where necessary.

Reply: We thank the reviewer for noticing this mistake and have changed all instances of C_w to Cw in the text.

Response to Reviewer 2

Reviewer Comment 1.12 — Just, as far I understand the ecology of the studied chronotypes, is "global" sympatry describing fully their spatial distribution, or could it refer to micro-parapatry?

Reply: An argument can be made that the contemporary distribution of larvae for the Roscoff chronotypes might be described as "mosaic micro-parapatry", as separation over depth in the intertidal zone has resulted in regions enriched for one chronotype or the other (see supplemental figure 5 from Ekrem et. al., 2024 doi: https://doi.org/10.1111/ele.70139 for a map of Roscoff with genotyped larvae). However, these small scale spatial differences are likely well within the dispersal range for the highly motile adults, meaning that difference in spatial location are expected to play an insignificant role when it comes to preventing mating between chronotypes. Mallet et al., 2009 (doi: 10.1111/j.1420-9101.2009.01816.x) defined sympatry as the case when individuals from two populations are physically capable of encountering one another with moderately high frequency. If it weren't for the difference in reproductive timing between chronotypes, we believe that the Roscoff chronotypes would be encountering each other regularly. We therefore argue that "sympatric" is the correct term in this case.

Reviewer Comment 1.13 — Also, the results are really convincing to favor allochronic divergence. However, the term speciation is sometime used in the text (for instance line 359), without clear explanation about when the authors consider that divergence lead to speciation. In other term, do they consider speciation as an ineluctable final step of the modelled divergence?

Reply: Our results suggest that genetic divergence is a common result of *C. marinus'* reproductive ecology when larvae compete for space, and this genetic divergence could lead to the chronotypes becoming "good species". However, we recognize that there are many factors involved in the speciation process (such as the evolution and coupling of different reproductive barriers, or adaptation to occupying separate niches) that were not included in our model. Therefore, we have scaled back the use of speciation in our results and instead refer to the splitting of the chronotypes only as "divergence" or "genetic divergence".

Reviewer Comment 1.14 — L16 The use of the adverb "However" sounds strange, as the second sentence introduces a concept not simply and directly in opposition or restriction to the first sentence of the abstract.

Reply: We thank the reviewer for pointing this out and have changed it in the text.

Reviewer Comment 1.15 — The paragraphs are easy to follow in this order, but the first paragraph of the "Results" (l 136-167) corresponds to the description of the biological case, which could be in the Introduction. In the same way, the following paragraph (l 168-213) corresponds more to an overview of Material and Methods than to Results.

Reply: We thank the reviewer for suggesting this clarification to the structuring. To address this point, we have shifted the paragraphs between lines 168 and 213 to the methods section. In the same spirit, we also moved moved much of the the paragraph describing the model extensions in the "Results: Adding Recombination and a Genetic Basis" section to the "Methods: Extended Model" section.

However, we left the more detailed description of the *C. marinus* system at the top of the results as those details feed directly into the decisions we made when devising our model and model assumptions. We thought they would be better placed in context with the model description and results, rather than in the introduction.

Reviewer Comment 1.16 — L 152 the sentence refers to Fig 1 top left (not bottom left).

Reply: We again think the reviewer for catching this mistake and have fixed it in the text.

Response to Reviewer 3

Reviewer Comment 1.17 — First, the authors provide strong justifications for most of their assumptions, but they do not explain the biological relevance of their emergence time assumption (line 154), beyond model simplicity. To strengthen the article, we recommend providing additional context.

Reply: We thank the reviewer for this suggestion, and have added some additional detail to the sentence on line 159 to provide context for that assumption.

Reviewer Comment 1.18 — Second, we recommend stating explicitly the type of simulations being used, in order to make the article more broadly informative to other researchers.

Reply: We have added a few sentences at the beginning of the Methods:Extended Model section where we are more specific about the simulations we used, particularly that they are forward-in-time genetic simulations and do not follow a Wright-Fisher model.

Reviewer Comment 1.19 — Third, to strengthen the literature review of allochronic isolation, we suggest noting the role of allochrony in the ecological divergence of both Rhagoletis fruit flies and their parasitoid wasps. In particular, we note the recent publication by Yee et al. (2025), "Concordance of eclosion life history timing across trophic levels in communities of host plants, fruit flies, and parasitoid wasps in the Pacific Northwest, USA."

Reply: We appreciate bringing this article to our attention. We are familiar with the Rhagoletis system, and though it provides one of the most convincing examples of speciation with gene flow accompanied by allochrony, according to the definition in Taylor and Friesen 2017 (DOI: 10.1111/mec.14126, see 2nd paragraph of right column on page 3332) the Rhagoletis system is not a case of allochronic speciation. This is because allochrony came about in conjunction with host-shifts, and thus allochrony could not be said to be the initial cause of divergence, which is a requirement of allochronic speciation. Since the intended goal of our literature review section was to highlight how examples of allochronic speciation are rare, we chose to focus solely on those cases, rather than cases of allochronic isolation in general. Therefore, we decided to exclude the Rhagoletis system in our review of allochronic speciation.
