# Supplementary material for: How competition can drive allochronic divergence: A case study in the Marine Midge, Clunio marinus
Source: PLoS Comput Biol. 2026 Apr 27;22(4):e1014235. doi: 10.1371/journal.pcbi.1014235 (PMC13148829; doi:10.1371/journal.pcbi.1014235)
Supplement: Attachment — Submitted filename: Jacobsen_et_al_PLOS-compbio_mating_types_2025_PEERREVIEW.docx [file pcbi.1014235.s005.docx]

This is an interesting manuscript of great biological relevance that would benefit from improving its structure, presentation and clarity.

This article develops an individual-based adaptive dynamics model to propose an alternative mechanism by which allochronic divergence in *C.marinus* could evolve; through evolutionary branching. I am pleased with the authors’ efforts to include a genetic basis to the model to account for ecological realism. The authors have been particularly thorough in their investigation. A range of different fitness functions and initial conditions were considered to ensure robustness of the model’s results.

The presentation and verbal exposition seem terse at places, and I have some crucial comments on notation and clarity, along with some suggestions for the authors to consider. I therefore recommend a minor revision and look forward to receiving a revised version of the manuscript.

**Minor comments:**

1. In line 195 where it states “an integer T which ranged from 0 to 30”., it’s clear that T refers to the emergence phenotype. Using T to denote both the timing of emergence phenotype and the lunar day may lead to some confusion. I suggest using something like T_e to denote the phenotype.
2. In lines 202-213, it states that “changes to the emergence phenotype could only occur if the random number drawn was greater than 0.5 or less than -0.5”. If μ=0.5 and σ=0.18, the chance of getting anything less than -0.5 is incredibly low, biasing mutations towards those that increase T. I suggest the authors to either reconsider the algorithm for generating mutations or refine their explanation for how the mutations are implemented, with a mention of its propensity for increasing/decreasing T.
3. I notice that mutations may act to increase/decrease the emergence phenotype T. Please could the authors explain how they handled the scenario where a mutation occurs at a boundary case i.e. if a mutation decreasing T at T=0 or increasing T at T=30. It would seem unreasonable if a mutation caused T to jump all the way from 0 to 30 or vice versa.
4. In Figure 3, the simulation is run for “500 generations” while in Figure 4, the simulation is run for “1500 generations” and 3000 generations in Figure 5. I would appreciate if the authors could explain why the simulation was run for a different number of generations in each model (clonal, sexual and sexual with divergence in genetic parameter space).
5. In the environmental fitness F_e in equation 2, please provide some sentences to justify the choice of the constant 6 in e^{6D+1} as well as the + 1.
6. Lines 422-428 in the discussion provides a description about the parameter values where divergence occurs, however the wording makes it seems confusing e.g “Cw = 0.25 to Cw=0.025 and $\sigma_{dispersal}$=1”. I had to refer to figure 4 to understand precisely what that meant. I recommend the authors to explicitly refer to figure 4 and rephrase it, for example “in the model with recombination (see figure 4), we observe divergence for values of (Cw,$\sigma_{dispersal})$ ranging from (0.25,0.25) to (0.025,1)”.

Alternatively, the authors may consider placing this description in the results section rather than keeping it in the discussion.

7. I understand that the current model is an individual based model. However, it would be worth mentioning somewhere in the discussion about the possibility of an alternative ODE based approach that enables some analytical reasoning (see Geritz et al 1996, Phys. Rev. lett. [10.1103/PhysRevLett.78.2024](https://doi.org/10.1103/PhysRevLett.78.2024), Liu et al 2025 Plos Comp Biol [Cell size and selection for stress-induced cell fusion in unicellular eukaryotes | PLOS Computational Biology](https://journals.plos.org/ploscompbiol/article?id=10.1371/journal.pcbi.1012418)). This opens up the possibility of identifying evolutionary attractors and evolutionary singularities that dictate such branching event. I realise that incorporating genetic recombination may make this challenging, but a few sentences to discuss the feasibility of such approaches would be valuable.
8. It took me a while to realise that $\sigma_{effect}$ is only present in the sexually reproducing diploids model, after seeing it in Table 2. Please make this clear in section Methods:Emergence.
9. Line 228, typo $\sigma_{divergence}$, please correct to $\sigma_{dispersal}$.
10. Cw is denoted as C_w in places e.g. on line 427. Please choose your desired convention and correct where necessary.
